# A short report on knowledge exchange through research-based theatre: ‘Inside out of mind’

**DOI:** 10.1016/j.socscimed.2014.07.049

**Published:** 2014-10

**Authors:** Justine Schneider, Stephen Lowe, Tanya Myers, Kezia Scales, Simon Bailey, Joanne Middleton

**Affiliations:** a School of Sociology & Social Policy, University of Nottingham, UK; b Meeting Ground Theatre Company, Nottingham, Nottinghamshire, UK

**Keywords:** UK, Ethnography, Theatre, Dementia, Carers, Ethnodrama

## Abstract

The short report describes the development from page to stage of a work of theatre based on an ethnographic study. The originating research focused on the work of health care assistants (nurse's aides) whose direct impact on the quality of life of highly dependent people is often overlooked. The research followed hospital personnel on wards specialising in the 'challenging behaviour' associated with dementia in central England. Conventional research outputs failed to engage the health care assistants themselves, so we turned to theatre to remedy this. The development of the field notes into theatre was characterised by the artistic freedom given to the playwright, in contrast to more data-led approaches to theatre making. The account of the process of creating the play, *Inside Out of Mind*, is followed a description of how the work was received by specialist and general audiences totalling 2000+. The discussion seeks to locate the whole enterprise in relation to the field of research-based theatre and explores how the production and its associated learning events relate to definitions of research-based theatre in the light of recent attempts to encapsulate this broad and diverse methodology.

## Highlights

• Theatre is a powerful tool for communicating research findings in health care.
• Expert and lay audiences welcomed a theatrical drama about health care assistants.
• Producing good drama proved compatible with engaging stakeholders effectively.
• The interactive nature of the theatre experience makes each performance unique.

## 1 Background

Research in health care seeks improvements overall but changing minds is not enough: research needs to act on how people feel about things as well as what they think, and in a complex health care environment this is particularly difficult because of the need to influence groups and organisational cultures as well as individuals. This report describes a project which aimed to influence health care workers caring for people with dementia by creating a piece of theatre, based on field notes gathered in the course of commissioned, qualitative research on dementia wards. Alzheimer's Disease and related disorders (which we refer to here as dementia) pose a major challenge with implications for how developed societies prioritise their health care budgets. The focus of the initial study was the work of health care assistants (HCAs) in England, called ‘nursing aides’ in some other countries (Schneider et al., 2010). These people typically have no professional registration but they often make up the majority of hands-on carers for people with dementia. HCAs have not been studied much except from a management perspective (Bach et al., 2008) but they are an area of growing concern (RCN, 2012). In England the job requires no formal qualifications, the working conditions can be stressful and there is seldom time for training or supervision. The initial study's purpose was to inform central UK policy and planning for that workforce (see disclaimer). The project presented here was conceived post-hoc and evolved incrementally as resources became available. The project was driven by the research team's conviction that the findings should be disseminated effectively to the workforce we had observed. We also believed that the working lives of these carers would be of interest to a wider audience, including the health service and the general public. We saw dramatization as a means to open up our findings and to embody them, complementing the formal publications and enabling new interpretations of the data.

### 1.1 Originating research

The research, designed as a multi-site mixed-methods study using participant observation, interviews and focus groups, recruited two Master’s-level and one postdoc researcher, authors KS, JM and SB, who worked as participant observers while also working as health care assistants on dementia wards. The aim of the study was to explore the stresses, coping strategies and rewards of caring for people with advanced dementia. Formal ethical approval was granted by Derby local research ethics committee on behalf of the national committee, COREC. Staff members were briefed by the chief investigator about the study and visitors were notified that the research was ongoing through posters on the wards. Staff could choose if they wished to have their data excluded from the analysis (none did so). It is important to note that direct observations of patients were not part of the study, so no consent was sought from them and the ethnographers observed staff, rather than patients or visitors to the wards. Obtaining informed consent from those who lack capacity and their carers is, like many aspects of dementia care, an ethically grey area. Our approach, approved by the ethics committee, enabled us to overcome this potential obstacle whilst still representing the reality of life for both patients and staff on the wards.

The researchers collected field notes at three sites over a total of four months' participant observation, they also interviewed 30 individuals altogether and conducted at least one focus group in each site. They produced about 600,000 words, but the report to the research funders had to be no more than 40,000 words long. This addressed key themes concerning the workforce: stress, ward management, involving family members, inter-professional working, positive and negative aspects of team identity, attachment and loss. However, it only permitted us to deal briefly with some of the most compelling data, partly because we were not formally observing patients and partly because this went beyond the purpose of the commissioned research. To convey the comedy and tragedy of life on the wards we turned to theatre.

### 1.2 Theatre and knowledge exchange

HCAs have few opportunities for professional development and were not likely to read our formal publications. Therefore the findings of the study were mainly restricted to an academic and policy-making audience that accessed them through conference presentations and formal publications (Lloyd et al., 2011, Scales et al., 2011, Bailey et al., 2013) with one article in a practitioner journal (Schneider, 2010). We felt an obligation to share the findings effectively with the people who had permitted us to observe their work, in the hope that this might help to improve dementia care – broadly, by enabling the practitioners to reflect on the job, increasing their awareness of the personhood of patients and of their own needs for recognition and respect. Feedback sessions by the research team held between shifts attracted only a handful of health care assistants, for reasons which were difficult to ascertain. Yet the field notes contained vivid, compelling and inherently dramatic accounts of the daily work of the wards, and we wanted to make this reality better known, both to HCAs themselves and to a general audience including other NHS personnel. We saw the re-enactment of a dramatized version of life on the ward as a means to make this closed environment widely accessible.

The research team intuitively recognised the superiority of drama over text when it comes to portraying the multidimensionality of human experience. In fact, led by pioneers (Mienczakowski, 1995, Denzin, 1997, Saldaña, 2005) and others, the ‘ethno-drama’ tradition has systematically gathered data for the express purpose of communicating the findings in theatrical forms. This has shed light on cancer (Sinding et al., 2002), homelessness (Finley and Finley, 1999) and racism (Goldstein, 2001) as well as dementia (Kontos and Naglie, 2007), with at least one study reporting a ‘shift’ in attitudes towards dementia on the part of health care personnel following attendance at a theatrical presentation (Jonas-Simpson and Mitchell, 2005).

Typically, ethno-drama begins with the intention of producing a dramatisation as a means to communicate research findings. In our case, this was an unplanned and unanticipated output, but one that responded directly to the field notes and was consistent with our felt obligations to engage the participants of our observations, health care assistants, with our findings. The implications of this response for bridging applied social research and the arts warrant investigation. We discuss them here in relation to a theoretical framework which has been put forward by Beck et al. (2011) to enable comparisons and differentiations to be made amongst divergent approaches to research-based theatre. Building on typologies of research-based theatre developed by Denzin (1997) and Rossiter et al., 2008, Beck et al., 2011 propose a two dimensional framework, with the axes ‘research’ and ‘performance’. This characterises ***research*** along a continuum with four reference-points: systematic research; more informal yet still first-hand research; second-hand research; and casual enquiry. The framework also offers four reference-points for ***performance***: researcher-only or closed performance; research stakeholder performance; aesthetic and stakeholder performance; and aesthetic performance. Having described the evolution of our research-based theatre project below, we will seek to locate it in relation to these points on the research-performance axes.

## 2 Methods

### 2.1 Commissioning

The script was commissioned from a local theatre company, Meeting Ground, through a contractual agreement with the university. This entailed a confidentiality agreement to give access to the field notes, which had been fully anonymised so that no individual could be identified. An Independent Theatre Company (ITC) contract defining rights with respect to the script and any productions was also made. Meeting Ground Theatre Company's experience and track record lie in what is called ‘politics of the imagination’, based on the principle that imagination is a route to understanding and insight into the worlds of others. Fortuitously, the theatre company founders had been influenced by the work of Augusto Boal, who founded the Theatre of the Oppressed, using drama to challenge injustice and raise the consciousness of participants (Boal, 1993), so their approach was to empower the research participants through drama.

Unlike some theatre commissions which have explicit educational objectives or messages to deliver, since the final report had been submitted and their contractual commitments to funders were met, the research team chose to give total freedom to the playwright, Tanya Myers. Support was provided by Dr. Stephen Lowe with responsibility for the artistic development, and steering the work to completion. This entailed giving the playwright and other creative artists (set, costume, sound and lighting designers as well as the director) freedom to explore the work's individual sense of form and beauty. Researchers and artists shared the conviction from the outset that drama generated through this creative and imaginative process would have the potential of reaching a wide general audience, provided it was unconstrained by ‘worthy’ or didactic aims that might make it instructive rather than inspiring.

### 2.2 The creative process

The script development took 14 months, during which playwright immersed herself in the field notes but also read many papers and books on dementia and drew on her own experiences of caring for people with dementia. Not without reservations, the researchers believed that, on balance, the potential advantages of effective knowledge transfer outweighed the risks, motivated by a conviction that the research report did not do justice to the extraordinarily vivid field notes.

## 3 Results

### 3.1 The characters and script

The resulting work, *Inside Out of Mind*, has 15 characters, including eight carers (of whom six represent paid professionals and one a family carer) and seven patients. Each of these characters has a past, present and future reality imagined by the writer. Staff carers and much of their dialogue came directly from the research material, while characterisation of the patients and their family members drew on wider reading and the writer's own experience of working as a carer and looking after a family member with dementia. As the paid carers were the focus of our observations and patients were not observed directly, the characterisation of the patients in the play is not based on field notes. Rather, the patients with dementia are fully the author's creation and include a former French Resistance fighter, a prima ballerina and a childless woman still traumatized by loss of her sister in a bus accident during a blackout in World War II. The script supplements the data collected through systematic participant observation with first-hand experience and second-hand sources. The patients are therefore wholly fictional, while the staff are composite characters based on the research data; no recognisable individual is portrayed.

### 3.2 Productions

In July 2011, following five days of workshops directed by Stephen Lowe, excerpts from the play were presented at Nottingham Playhouse to an invited audience of about 60 professionals from health, theatre and research backgrounds, together with family carers of persons with dementia, with a panel discussion to follow. A 10-min film about the workshop can be viewed here: http://vimeo.com/32860587 or as supplementary material. The audience response encouraged the team to work towards a full production. Bringing this to the stage took two years, two rewrites of the script and several bids for grant funding (three unsuccessful, and one successful). Three local health care providers agreed matched funding. Most important to the communication of the drama to our intended audience, directors of nursing and workforce development, who together employ about 1500 health care assistants, were persuaded to make a commitment to release most of these members of staff to attend a performance of the play. Moreover, three health care organisations seconded senior nurse educators for 30 days over 9 months to ensure that each organisation and its staff were fully prepared for this to happen. The university arts centre made its theatre available for two weeks in June, 2013, and its staff took charge of marketing and catering. Their guarantee of a venue enabled us to give the theatre company the green light to contract the professional actors, build the set and book rehearsal space.

Supplementary data related to this article can be found online at http://dx.doi.org/10.1016/j.socscimed.2014.07.049.

The following are the Supplementary data related to this article:

Video

### 3.3 Workshops

The assurance of an audience of health care assistants (who had been the main focus of the study) offered the opportunity to complement their attendance at the play with a linked workshop, completing the cycle of knowledge exchange, and making a structured learning event available to these basic-grade personnel who were rarely released for anything other than mandatory training. The production team drew on the enthusiasm and goodwill of 15 university lecturers and researchers in health to facilitate the workshops. These were planned to a high level of specification so that each participant would have a similar experience. The aim was formulated as follows: *The purpose of the Inside Out Learning Event is to increase people's confidence in caring in clinical settings for people with memory problems and the family members who support them*. A further opportunity to create an effective learning experience for the participants was offered by the Arts Council, Grants for the Arts funding to employ actors to co-facilitate each workshop. Thus the actors could demonstrate how they used imaginative skills to engender compassion - role-playing, hot seating and other professional techniques, and how these could be relevant to participants' experience of dementia care. The workshops were evaluated by the first author using three methods: brief questionnaires completed by participants on the day, verbal and written reflections made by the workshop leaders, and a focus group of the actors who co-facilitated which was recorded and transcribed for thematic analysis. A paper on the workshops and their evaluation is in preparation. The fact that participants completed the questionnaires was taken to imply informed consent, while the workshop facilitators were recruited and prepared on the understanding that they were collaborating in a developmental process entailing a formal evaluation.

### 3.4 Full production

The playwright herself (Author TM) directed the full production at Lakeside Arts Centre, University of Nottingham, in June 2013, realising her vision for an elaborate, complex and sometimes surreal treatment of the script. The developed play includes visual and auditory themes, objects as symbols, dance and songs from the 1940s. Books, trees, birds, loud noises and keys are just some examples of imagery that reveals multiple layers of significance within the performance. The Nottingham production is uncompromising in terms of aesthetics: the audience enters directly into the noisy, confusing and impenetrable world of a person with dementia, and much of Act One is only interpretable as the story unfolds. The transition from bewilderment in Act One to recognition in Act Two mirrors the confusion of a person with dementia: this is one example of the many layers of meaning that the play offers attentive audience members.

Fifteen performances were given in June 2013; eight of these were exclusively for audiences of health care assistants, each one followed by a discussion between the cast and the audience, who then participated in 2-h workshops. 1109 health care assistants attended the learning events and 863 (78%) completed evaluation forms). Of this number:

- 52% said they had received no training of any kind in relation to dementia although employees of a specialist mental health trust were significantly more likely to have had training in dementia than those who worked in an acute trust;
- 82% said they worked with people with dementia at least once per month; and
- 92% said the day would have a positive impact on their work with people with dementia.

The vast majority of the health care assistants (84%) had not been to a theatre in the past year, some had never been to see a play before. We were especially gratified when the novices demonstrated a quick understanding of the subtleties of the play. Some brought their families back to see one of the public performances. Not all enjoyed the workshops, and a few could not overcome their discomfort about participating in role play – but 93% rated the day ‘good’, ‘very good’ or ‘excellent’ overall. In the audience discussions, each chaired by a senior executive in the employer trusts, participants thanked the writer and validated the play's presentation of their working lives, while recognising that the dramatisation frequently deviated from their literal reality. Participants also challenged the status quo: why they generally receive so little recognition for the work they do. *Inside Out of Mind* shows them under stress, underappreciated and underpaid; human beings with feelings and families who grow attached to their charges at work.

There were 75 written comments and 24 of these were negative (3% of all respondents). Nine negative comments came from the first day, when the conference registration process and other logistical problems were to blame. Overall, written feedback supported the quantitative evidence that the workshops achieved their aim of increasing people's confidence to care, with 92% stating that the event would help them in working with people with dementia. Comments reflect a positive appreciation of the innovative approach to education using theatre: ‘brilliant’ and ‘amazing’ were often used. They show the emotional impact of the performance:

“*I have learnt that people with dementia have feelings and I have learnt to appreciate how they must feel*.”

Respondents also endorsed the relevance of the show for a much wider audience.

“*Excellent for giving an insight to people unaware of dementia and what can be experienced. Should be shown nationwide*.”

A seven-minute film about the play's immediate impact can be viewed here: http://www.youtube.com/watch?v=Kbir0cCQPYM.

This includes brief scenes from the play and comments from members of the team, together with interviews with three HCAs who express the enthusiastic response shown in the formal evaluation.

## 4 Discussion

Here we address the methodology of research-based theatre in this context, while analysis of the lasting impact of the play awaits follow-up data. Our preliminary findings tend to confirm an earlier report of the ‘shift’ made by health care professionals who attended a research-based play about living with dementia (Jonas-Simpson et al., 2012). This effect can be described as inspiring greater compassion, meaning and hope concerning dementia work. Anecdotally, people who saw the play several times say they took away a different understanding on each occasion.

How, then to characterise the play and its relevance to the field of social science? The Beck et al. (2011) dimensions of ‘research’ versus ‘performance’ emphasis places the preliminary performance of *Inside Out of Mind* and its full production in different positions. The early performance of excerpts was clearly a combination of systematic research (field notes) first hand informal research (playwright's lived experience) and second-hand research (playwright's reading), and it was presented to a stakeholder-only audience. The full production likewise spans three fields of the research spectrum: systematic research, lived experience and second-hand experience, but the performance was presented to eight audiences of stakeholders and seven audiences made up of the public – albeit with a bias towards academics and health care professionals among the ‘public’. It could therefore be classed as an ‘aesthetic/stakeholder performance’ or, given the elaborate staging, lighting and soundscape, it might lean towards the aesthetic performance. Moreover, different treatments of the script and different audience profiles might lead us to position the work in different locations on the research-performance framework.

In attempting to apply the framework proposed by Beck et al. we find that we need three, not two dimensions, because ‘audience’ and ‘aesthetic’ are confounded in the present formulation and in practice they vary independently. *Inside Out of Mind* demonstrates that aesthetic values do not have to be compromised in order to engage stakeholder audiences, it therefore challenges the reciprocal relationship between audience and aesthetics as portrayed in Beck et al.’s typology. Nonetheless it should be noted that for the stakeholder performances we found it necessary to explain in advance to the audience of health care assistants that the world they were about to enter was that of a person with dementia. Without this contextual information the experience proved inaccessible to many.

Perhaps research-based theatre defies categorisation; in many ways the product is unique, each time it is performed. The translation of research into drama entails a dialogue between the data and the script; numerous judgements are made – mainly by the writer, to edit and elaborate an already-selective research account of observations. This is followed by a further process of transformation of the script, inspired by the director's vision, but affected also by the actors' physicality and characterisation, the physical environment or set, and finally by the audience as well. Judgements about research data are guided by the values, history, standards and politics of research, while the decisions of the writer, director and actors are guided by the values, history, aesthetics and politics of theatre, and these are not static. It is important also to remember that each audience is different, and that actors respond subtly to the audience reactions; this was particularly evident in our experience when the theatre was full of those health care professionals whose working life was being portrayed on stage.

Research-based theatre seems to be governed by a general rule, expressed pithily by Beck et al.: ‘[t]heatre created to disseminate findings may seek to be more objective than compelling, while theatre created for the general public may seek to be more compelling than objective.’ [24, 688] The success of *Inside Out of Mind* might not have been achieved if the project had been undertaken as ‘strict’ ethno-drama from the start; a commission whose intention was principally to portray research findings (rather than to achieve knowledge exchange with the target audience) would have required a more direct representation of the data collected, and would probably have attracted a narrower audience of insiders. As a team we were not burdened by prior assumptions about research-based theatre and the stringencies of ethno-drama, the aesthetic held sway over the realistic because we unhesitatingly subscribed to the objective of reaching as large an audience as possible. In other words, *Inside Out of Mind* appears to defy the general rule that places compelling theatre and impact on stakeholders in opposition to each other. It therefore indicates that the Beck, Belliveau et al. framework should be revised to accommodate a third dimension, differentiating ‘audience’ from ‘aesthetic’, because we have found that sizeable numbers of stakeholders can engage with a performance that prioritises artistic merit.

As we see it, deployed as a methodology in social research, ethno-drama is dialogue between the ethnographic and the dramatic. The ethnographic entails the documentation of a social setting, and the dramatic entails its re-enactment – generally not (perhaps never) a literal reproduction, but essentially one based on key emotional themes arising in that observed setting. To that extent, the term ethno-drama can be applied to *Inside Out of Mind*, and the analysis of the reality observed by the study's ethnographers continues to this day in the minds of the audience, in further performances of the play and in ongoing responses to the associated learning events.

## 5 Conclusion

“Theatre has a unique potential to interpret, translate and disseminate research findings. This is especially true for medical and health-related knowledge, which often revolves around complex questions of the embodied human condition and which … often fails to inform those outside academic settings …” (Rossiter et al., 2008, 131). Live theatre presentation of observation-based dementia care to the subjects of our observations and people like them has proved to be a powerful medium which has seldom before been used on such a scale. The translation of research into a high-quality theatre production proved successfully to engage its target audience, health care assistants working in dementia. Feedback from this audience demonstrates that *Inside Out of Mind* has potential to change hearts as well as minds and is an effective tool for reflection. Follow-up evaluation over a longer time-frame will show whether it has a lasting impact; whether it inspires change or empowerment in those who see the play. Our impression from radio and newspaper coverage is that the play's portrayal of the reality of dementia care also successfully engages the imaginations of the ‘lay’ audiences who have seen it so far, raising awareness about the issue of dementia and its implications for society as a whole, while respected authorities in the arts affirm the production's artistic merit. This play therefore demonstrates the power of theatre to translate and communicate health-related knowledge to expert and lay audiences alike. Unlike a published research paper, however, the play will continue to change with each performance and each new audience, developing organically and interactively. Given the dynamic nature of theatre, there is no way to control or prevent this evolution, we can only observe what remains constant and what changes.

## Disclaimer

The views and opinions expressed herein are those of the authors and do not necessarily reflect those of the NHS, the NIHR, the HS&DR/SDO programme or the Department of Health.

## Funding statement

The originating research was supported by UK NIHR SDO/HS&DR grant number 01/1819/222.

## Competing interests

None declared.
